# *N*-Butanol Extract of *Gastrodia elata* Suppresses Inflammatory Responses in Lipopolysaccharide-Stimulated Macrophages and Complete Freund's Adjuvant- (CFA-) Induced Arthritis Rats via Inhibition of MAPK Signaling Pathway

**DOI:** 10.1155/2020/1658618

**Published:** 2020-01-21

**Authors:** Peng He, Yiwen Hu, Changzhao Huang, Xi Wang, Heng Zhang, Xianping Zhang, Houjie Dai, Ruiying Wang, Yan Gao

**Affiliations:** ^1^Department of Orthopedics, Affiliated Hospital of Guilin Medical University, 15 Lequn Road, Guilin 541001, China; ^2^Department of Orthopedics, Huludao Central Hospital, 15 Lianshan Street, Huludao 125000, China; ^3^Nursing College, Affiliated Hospital of Guilin Medical University, 15 Lequn Road, Guilin 541001, China

## Abstract

*Gastrodia elata* is a traditional herbal medicine that has been used for centuries to treat rheumatism. Previous studies have confirmed that ethanol extracts of *Gastrodia elata* have anti-inflammatory and antioxidant activities, and the *n*-butanol fraction exerts a higher inhibitory effect. However, the in vivo anti-inflammatory effects of *Gastrodia elata* have not been evaluated. Thus, we assessed the therapeutic effect of the *n*-butanol extract of *Gastrodia elata* (BGE) on complete Freund's adjuvant- (CFA-) induced arthritis rats which were separated into six groups (NOR; MODEL; CFA + dexamethasone (DEX); CFA + 25, 50, 100 mg/kg BGE). The paw swelling, joint radiology, and histology were used to analyze the effect of BGE on delaying the progression of rheumatoid arthritis. Furthermore, serum levels of inflammatory cytokines were analyzed via ELISA. In addition, the effect of BGE on nitric oxide (NO) production, expression of inducible nitric oxide synthase (iNOS) and cyclooxygenase-2(COX-2), and inflammatory cytokines were detected in lipopolysaccharide- (LPS-) stimulated RAW264.7 macrophage cells. Lastly, the impacts of BGE on the activation of the mitogen-activated protein kinases (MAPK) pathway in CFA rats and LPS-stimulated RAW264.7 macrophage were examined by western blot analysis. The results show that BGE can significantly reduce paw swelling without losing the body weight of rats. Imaging assessment confirms that BGE can protect cartilage from destruction, as well as reducing inflammatory cell infiltration and synovial proliferation. Moreover, BGE suppresses the production of inflammatory cytokines in serum and inhibits the activation of the phosphorylation of p38 and ERK in CFA rats. BGE was also demonstrated to decrease the production of NO and inflammatory cytokines in LPS-stimulated RAW264.7 cells. The effect of BGE in LPS-induced expression leads to reduced p38 and ERK phosphorylation and also downregulates the protein expression of iNOS and COX-2. Taken together, BGE exhibits a potential therapeutic effect on CFA rats, and its anti-inflammatory and antioxidant effects were possibly exerted by regulation of ERK/p38MAPK.

## 1. Introduction

Inflammation is a complex defense mechanism. In general, the inflammatory response is beneficial to the body, by neutralizing and restoring normal functions of cells and tissues. But sometimes, inflammation is also harmful, such as some diseases that attack the body's own tissues [1]. Rheumatoid arthritis (RA) is a systemic autoimmune disease characterized by polyarthritis, progressive joint damage, and joint deformities. The pathological features of RA are joint synovial lesions, including hyperplasia of synovial cells, infiltration of inflammatory cells, formation of vasospasm, and destruction of joint bones and cartilage. Patients eventually lose joint function due to joint stiffness, which seriously affects their quality of life [2–4]. According to relevant research reports, the incidence of RA in the world is about 0.5–1% [5]. If RA is not properly treated, it can rapidly develop into multiple systems of inflammation and irreversible joint damage, causing disability and premature death. Because the etiology and pathogenesis of RA are not clear, there is no effective treatment.

While the pathophysiological mechanisms of RA remain unclear, it is known that various proinflammatory mediators and cytokines are involved in RA pathogenesis [6]. The generation of hypertrophied synovial involves infiltration of macrophages and their interaction with fibroblasts translates into an enhanced presence of proinflammatory cytokines, particularly IL-1*β*, IL-6, and TNF-*α* [7]. Eventually, the interaction between these proinflammatory mediators leads to synovial inflammation, cartilage damage, and bone destruction. Accordingly, the therapeutic strategies include approaches that disrupt recruitment of inflammatory cells and limit the proinflammatory. Mitogen-activated protein kinase (MAPK) is a highly conserved serine protein kinase in the cytoplasm that plays an important mediating role in the signaling of many cytokines. ERK and p38 as major members of the MAPK family have been confirmed to be related to the pathogenesis of RA [8]. p38 is an important intracellular signaling pathway that regulates the expression of cytokines such as IL-1*β*, IL-6, and TNF-*α* [9]. Experiments have confirmed that ERK plays an important role in mediating osteoclastogenesis and fibroblast-like synoviocyte proliferation, indicating that modulation of these signaling pathways are promising therapeutic targets for RA [10].

At present, the clinical treatment of rheumatoid arthritis drugs mainly include nonsteroidal anti-inflammatory drugs (NSAIDs), disease-modifying antirheumatic drugs (DMARDs), steroid hormones, and biological agents (TNF-*α* antibody and TNF-*α* receptor inhibitor) [11]. These drugs have played an important role in the history of the treatment of RA, and some drugs are still used as clinical first-line drugs. However, long-term use of these drugs can lead to immunocompromise, bone marrow suppression, liver and kidney function damage, gastrointestinal dysfunction, and cartilage degeneration [12]. Although TNF-*α* inhibitors are currently the most used biological agent in the treatment of RA, they may lead to infection [13]. Therefore, it is urgent to find effective drugs with low toxicity to treat RA. In recent years, increasing evidence shows that traditional Chinese medicine can become a new strategy for the treatment of RA [14–16]. With the progress of traditional Chinese medicine research, a large number of pharmacological studies have shown that the alkaloids, flavonoids, and glycosides in Chinese medicine have analgesic and anti-inflammatory effects [17–19].

*Gastrodia elata* (Orchidaceae) is a valuable traditional Chinese medicine with high medicinal value. It is mainly distributed in plateau mountains in China, Japan, North Korea, and India. For centuries, *Gastrodia elata* has been used as an anticonvulsant, analgesic, and sedative, especially in the treatment of diseases such as epilepsy, dizziness, headache, rheumatism, neuralgia, and paralysis [20]. Until now, many chemical components have been isolated from *Gastrodia elata*, such as gastrodin, phenolic compounds, glycosides, polysaccharides, sterols, flavonoids, and organic acids [21]. The neuroprotective effect of *Gastrodia elata* has been confirmed by many studies. Many of the active ingredients extracted from *Gastrodia elata* have been used clinically to treat headaches, neurodegenerative diseases, dizziness, and epilepsy, and the treatment effect is remarkable [20, 22]. However, the pharmacological effects of *Gastrodia elata* have been widely reported in neurology. However, in the study of the neuroprotective effect of *Gastrodia elata*, some anti-inflammatory and antioxidative activities have also attracted the attention of scholars. For example, the phenolic compounds isolated from *Gastrodia elata* can significantly alleviate asthma symptoms. 4-Hydroxy-3-methoxybenzyl alcohol significantly reduces the specific airway resistance in asthmatic diseases and inhibits the increase in the number of eosinophils and neutrophils [23]. The mechanism of anticonvulsant effect of *Gastrodia elata* is that the main components of *Gastrodia elata*, 4-hydroxybenzyl alcohol, and other phenolic compounds (such as vanillin) have antioxidant effects [24].

In the 1990s, *Gastrodia elata* extract pill developed by Jiegu Zhang relieved the clinical symptoms caused by rheumatoid arthritis, degenerative osteoarthritis, sciatica, and gout. Previous studies have confirmed that the ethanol extract of *Gastrodia elata* can inhibit inflammatory response in fibroblast-like synoviocytes by inhibiting the NF-kB signaling pathway [25]. In addition, the ethanol extract of *Gastrodia elata* can reduce the increase in vascular permeability induced by acetic acid and can exert an analgesic effect in acetic acid-induced active writhing experiments, and the *n*-butanol fraction exerts the higher inhibitory effect [26]. To further investigate whether BGE can relieve arthritis symptoms, protect the cartilage and inhibit the inflammation in CFA rats. In our study, we evaluated BGE effects and explored the preliminary mechanisms by assessing joint swelling, cartilage damage, serum-related inflammatory factors, and tissue protein expression levels in rats with different doses of BGE. In addition, in order to understand the anti-inflammatory mechanism of BGE, we used ELISA and western blot analysis to analyze the changes of related protein pathways and inflammatory factors in LPS-stimulated RAW264.7 macrophages. Therefore, our results can provide evidence for the ability of BGE to delay the progression of RA.

## 2. Materials and Methods

### 2.1. Preparation of BGE

High-quality *Gastrodia elata* (qualified number 20180410YY) was purchased from Nanjing Herb Source Biotechnology Co., Ltd (Nanjing, Jiangsu, China). The dried *Gastrodia elata* (1 kg) was ground into powder using an electronic pulverizer. Then, soak it in ethanol (50.0%, 25 L) for 4 days. The crude extract was filtered through a qualitative filter paper loop and then concentrated by a rotary evaporator (Model: RE5203, Shanghai Yarong Biochemical Instrument Factory, Shanghai, China). Finally, the ethanol extract of *Gastrodia elata* (126 g) was obtained. The ethanol extract of *Gastrodia elata* was added to distilled water and suspended uniformly and extracted with petroleum ether, ethyl acetate, and *n*-butanol, respectively. The extracts of different polarities were passed through a rotary evaporator to finally obtain an *n*-butanol fraction (13.3 g) for subsequent experiments [26, 27].

### 2.2. Animals

Specific pathogen-free grade Sprague-Dawley rats (weighing 200–220 g, male) were purchased from Hunan Slack Jingda Experimental Animal Co., Ltd (Changsha, Hunan, China); animal license number: SCXK (Xiang) 2016-0002. All animal experiments were performed with the protocol approved by the Animal Care Welfare Committee of Guilin Medical University.

### 2.3. Complete Freund's Adjuvant (CFA) Model and Administration

Eight rats were randomly selected as the normal group. The remaining 40 rats were injected into the left hind paw with complete Freund's adjuvant 0.1 mL (Sigma Chemical Co., St Louis, MO, USA) after sterilization [28, 29]. Rats in the normal group were injected with the same amount of normal saline at the same site. Since day 14, 40 CFA rats were randomly selected and divided into 5 groups (8 rats for each group): model group (distilled water treatment), positive control group (0.4 mg/kg·d dexamethasone), low-BGE group (L-BGE: 25 mg/kg·d BGE), medium-BGE group (M-BGE: 50 mg/kg·d BGE), and high-BGE group (H-BGE:100 mg/kg·d BGE). The abovementioned animal BGE administration concentrations were determined by preliminary experiments. On the 15th day after immunization of the rats, CFA rats were treated by drug gavage for 15 days. The model group and normal group were given to distilled water in the same volume with rats in the treatment group.

### 2.4. Rat Paw Swelling, Body Weight, and Sample Collection

The degree of joint swelling was measured on days 0, 1, 2, 5, 8, 11, 14, 17, 20, 23, 26, and 29 after immunization. The toe volume of the inflammation side (left hind paw) of the rat was measured using a rat paw volume plethysmometer (Model: 37140 Ugo Basile, Italy), and the result was expressed as the volume of water displaced below the left ankle joint [28]. Rats were weighed once every 5 days after immunization. Six hours after the gavage on the 29th day, the rats were anesthetized by intraperitoneal injection of 10% chloral hydrate and blood was taken from the abdominal aorta. After rats were sacrificed (decapitation under chloral hydrate), the left ankle joints were separated, partially stored in a −80 degree refrigerator, and partially immersed in 4% neutral paraformaldehyde (Solarbio, Beijing, China).

### 2.5. Macroscopic Observation and Radiographic Analysis

At the end of the experiment, the rat's left hind paw was photographed with a digital camera. After anesthesia, the rat hind paws were photographed using a hand-held X-ray fluoroscopy (Model: BJI-1, Shanghai Boxun Medical Bio Instrument Co., Ltd, Shanghai, China). Set the portable X-ray fluoroscopy mode to medium and then adjust the background grayscale value for filming.

### 2.6. Histological Assessment

The left hind paw of the rat was immersed in 10% neutral paraformaldehyde for 24 h and then decalcified with ethylenediaminetetraacetic acid (EDTA) for 2 weeks. After the joints were gradient dehydrated by ethanol and dipped in wax, they were embedded in paraffin. Each specimen was cut into 6 *μ*m thick tissue sheets. Pathological changes in joint tissues were observed by hematoxylin-eosin staining (ZSHB-BIO, Beijing, China). Pathological sections were analyzed by the K-Viewer digital pathology system (version 1.5.2.5, Ningbo Jiangfeng Biological Information Technology Co., Ltd, Ningbo, Zhejiang, China).

### 2.7. Concentration of Cytokines in Serum

Rat blood was allowed to stand at room temperature for 2 h and then centrifuged at 1,377 ×g for 10 mins. Serum was collected from the upper layer of the centrifuge tube and stored in a −80 degree refrigerator. Serum levels of IL-1*β*, IL-6, and TNF-*α* were determined by enzyme-linked immunosorbent assay (ELISA) kits (Shenzhen Dayou Biological Engineering Co., Ltd, Shenzhen, China) in strict accordance with the manufacturer's instructions. The concentrations of IL-1*β*, IL-6, and TNF-*α* were calculated from the standard curve.

### 2.8. Cell Culture

The RAW264.7 murine macrophage cells (Shanghai Institute of Cell Biology, Shanghai, China) were cultured in high-glucose DMEM medium containing 10% fetal bovine serum, 100 U/mL penicillin, and 0.1 mg/mL streptomycin. The culture environment of the cells was 37°C with 5% CO2.

### 2.9. Cell Viability Analysis (CCK-8 Assay)

Cell viability was determined using a Cell Counting Kit-8 (Dojindo, Kumamoto, Japan). The cells were seeded at a density of 4 × 10^3^ cells/well in 96-well plates and cultured for 12 h at 37°C with 5% CO2 and then were treated with BGE (10.0, 20.0, 40.0, and 80.0 *μ*g/mL) for 20 h. After 20 h, CCK-8 was added to a 96-well plate (100 *μ*L per well plus 10 *μ*L CCK-8) and incubated at 37°C for 2 h. The absorbance at 450 nm of each well was determined by an enzyme-labeled instrument (Model: M200 PRO NanoQuant, Switzerland). The survival rate of each group was calculated according to the formula.

### 2.10. Measurement of NO, TNF-*α*, and IL-6 Levels in Cell Culture Supernatants of LPS-Stimulated RAW264.7 Cells

RAW264.7 cells were seeded at 2 × 10^5^/well in 6-well culture plates. After 12 h of culture, the cells were treated with varying concentrations of BGE (10.0 *μ*g/mL, 20.0 *μ*g/mL, and 40.0 *μ*g/mL) and Dex (10^−4^M), followed by stimulation with LPS (1.0 *μ*g/mL, Solarbio, Beijing, China) for another 24 h. After 24 h, the cell culture supernatant was collected. The secretion of IL-6 and TNF-*α* and the amount of NO secretion in the supernatant were determined by ELISA kits (Shenzhen Dayou Biological Engineering Co., Ltd, Shenzhen, China) and the NO assay kit (Beyotime, Beijing, China) in strict accordance with the manufacturer's instructions.

### 2.11. Western Blot Analysis

The rat ankle joint was ground to a powder in liquid nitrogen. Protein lysate and phosphatase inhibitor were added to the tissue and allowed to stand on ice for 30 mins. Total protein was extracted after centrifugation at 4°C for 30 mins. Proteins were quantified using the BCA Protein Assay Kit (Beyotime, Beijing, China).

RAW264.7 cells were seeded at 2 × 10^5^/well in 6-well culture plates. After 12 h of culture, the cells were treated with varying concentrations of BGE (10.0 *μ*g/mL, 20.0 *μ*g/mL, and 40.0 *μ*g/mL) and Dex (10^−4^M), followed by stimulation with LPS (1.0 *μ*g/mL) for another 24 h. Protein lysate and phosphatase inhibitor were added to the tissue and allowed to stand on ice for 10 mins. Total protein was extracted after centrifugation at 4°C for 10 mins.

Total protein was separated by SDS-PAGE gel electrophoresis. The protein was transferred to a polyvinylidene difluoride (PVDF) membrane, and the membrane was blocked with 5% skim milk. Monoclonal antibodies directed against p-38, p-p38, ERK, p-ERK, iNOS, COX-2 (Cell Signaling Technology, Danvers, MA, USA), and *β*-actin (Proteintech Group, Rosemont, USA) were used to detect phosphorylated and total proteins. Following incubation with the primary antibody for 12 h, the PVDF membrane was washed three times for 10 mins with TBST and then incubated with a second antibody (Proteintech Group, Rosemont, USA) for 1 h. The hypersensitive luminescent liquid (Bridgen, Beijing Quanpin Biotechnology Co., Ltd., Beijing, China) is dropped onto the membrane, which illuminates under a gel imaging system (Bio-Rad, Hercules, CA, USA).

### 2.12. Statistical Analysis

Data were statistically analyzed by statistical program SPSS version 22.0, and the data were expressed as mean ± standard deviation. The comparison between the multiple groups uses one-way ANOVA, followed by Dunnett's test to detect intergroup differences. *P* < 0.05 was considered to be statistically significant.

## 3. Results

### 3.1. Effects of BGE on RA Symptoms and Body Weight in CFA Rat

**Figure 1:**
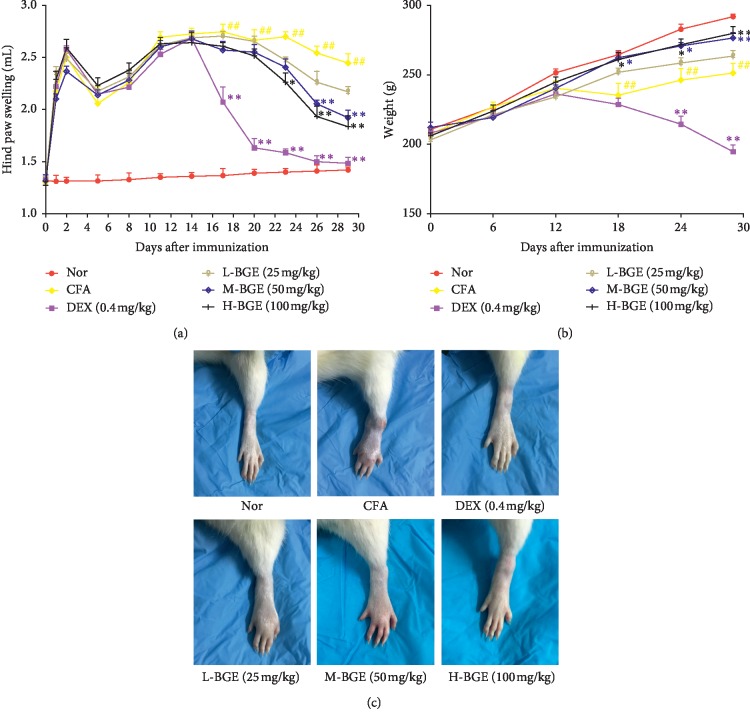
Antiarthritic effect of different doses of BGE in complete Freund's adjuvant-induced arthritis (CFA) rats. (a) Effects of different doses of BGE on hind paw swelling in CFA rats. Data were presented as mean ± SD (*n* = 8). (b) Effects of different doses of BGE on body weight changes in CFA rats. Data were presented as mean ± SD (*n* = 8). (c) Swelling manifestations in CFA rats after various treatments. Except normal group (Nor), CFA rats were treated daily with BGE at 25, 50, and 100 mg/kg·d^−1^, DEX at 0.4 mg/kg·d^−1^, or distilled water (CFA group) from day 14 after immunization. ^##^*P* < 0.01, compared with the normal group. ^*∗∗*^*P* < 0.01, ^*∗*^*P* < 0.05, compared with the CFA group.

As shown in Figure 1(a), the paw swelling of CFA rats reached a peak within two days after immunization. Subsequently, the swelling volume of the rat paw began to decrease, and the swelling volume reached its peak again from the 5th day to the 11th day. However, the arthritis symptoms and change of hind paw swelling volume reduced significantly after treatment with BGE (50 and 100 mg/kg) on days 23 and 26 (*P* < 0.01) (Figures 1(a) and 1(c)). Although treatment with dexamethasone for 6 days significantly ameliorated the symptoms of arthritis in rats, the side effects of dexamethasone caused a severe decrease in body weight in rats (Figures 1(b)). Compared with the model group, BGE can not only significantly improve the joint symptoms of rats but also effectively improve the slow weight gain of rats caused by immunization (*P* < 0.01).

### 3.2. Effect of BGE on Joint and Tissue Damage

**Figure 2:**
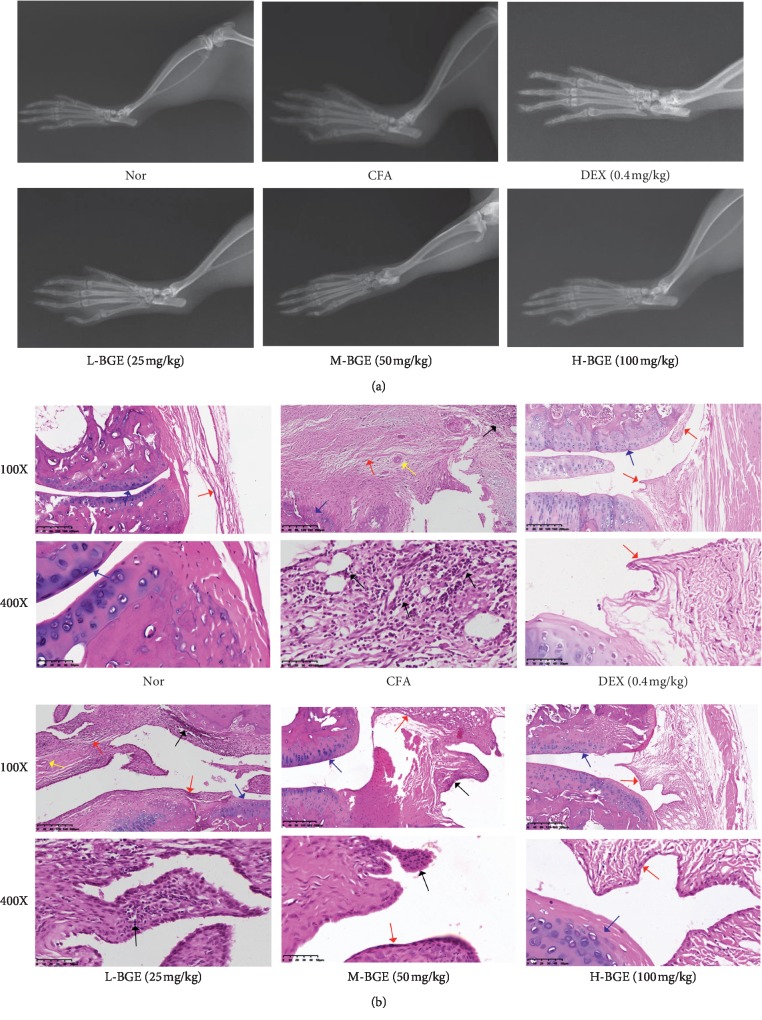
Radiological and histological evaluation of joint injury in CFA rats. (a) Representative X-ray images obtained from the toes and ankles of each group. (b) H&E staining of the ankle joint of CFA rats treated with various doses of BGE. Blue arrow, cartilage; black arrow, inflammatory cell infiltration; red arrow, synovial hyperplasia; yellow arrow, pannus.

In the model group, CFA rats showed obvious joint soft tissue swelling, joint space stenosis, and articular surface blur by radiographic analysis (Figure 2(a)). BGE can reduce the swelling of the soft tissue of the paw in a dose-dependent manner. Importantly, the joint space stenosis was improved in rats treated with BGE, which can be found by radiology. Compared with the normal group, the pathological sections of the ankle joint of CFA rats showed severe cartilage destruction, synovial hyperplasia, vasospasm formation, and a large amount of inflammatory cell exudation. Rats treated with dexamethasone showed only mild synovial hyperplasia and no significant inflammatory cell exudation and cartilage erosion. Compared with the model group, the BGE-treated (50 and 100 mg/kg) rats had significantly reduced the synovial layer and inflammatory cells, and the cartilage surface was smooth (Figure 2(b)).

### 3.3. Effect of BGE on the Secretion of Inflammatory Cytokines in Rat Serum and LPS-Induced RAW264.7 Cells

**Figure 3:**
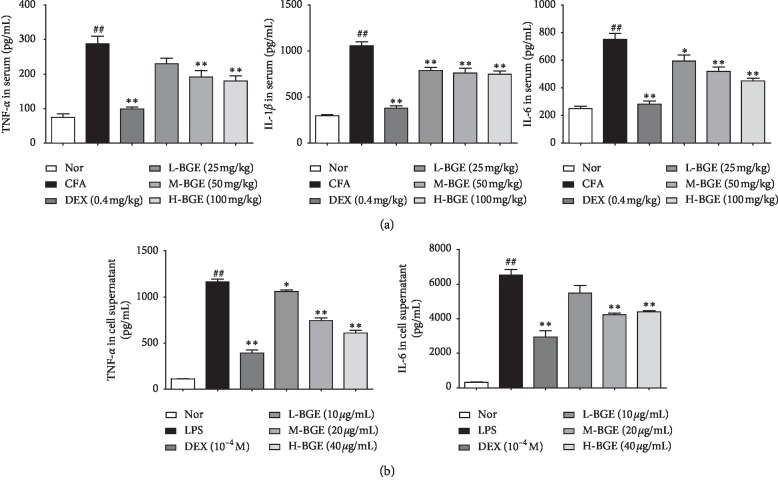
Effects of different doses of BGE on the expression of inflammatory cytokines in cell supernatant and CFA rat serum. (a) The concentrations of TNF-*α*, IL-1*β*, and IL-6 in serum were determined by ELISA. Data were presented as mean ± SD (*n* = 4-5). (b) The concentrations of TNF-*α* and IL-6 in cell supernatant were determined by ELISA. Data represent the mean ± SD of three independent experiments. ^##^*P* < 0.01, compared with the normal group. ^*∗∗*^*P* < 0.01, ^*∗*^*P* < 0.05, compared with the CFA/LPS group.

In patients with RA, elevated levels of IL-1*β*, IL-6, and TNF-*α* are associated with disease severity [30]. This condition is also evident in CFA rats (Figure 3(a)). The serum levels of IL-1*β*, IL-6, and TNF-*α* in the model group were significantly higher than those in the normal group (*P* < 0.01). BGE reduces the concentration of IL-1*β*, IL-6, and TNF-*α* in rat serum in a dose-dependent manner (*P* < 0.01). Similarly, dexamethasone also significantly reduced the levels of IL-1*β*, IL-6, and TNF-*α* in rat serum. In our experiments, the effect of BGE on proinflammatory cytokines in vitro was also explored. BGE (20 and 40 *μ*g/mL) can inhibit the secretion of IL-6 and TNF-*α* in LPS-stimulated RAW264.7 macrophages (*P* < 0.01) (Figure 3(b)).

### 3.4. Effect of BGE on RAW264.7 Cell Viability

**Figure 4:**
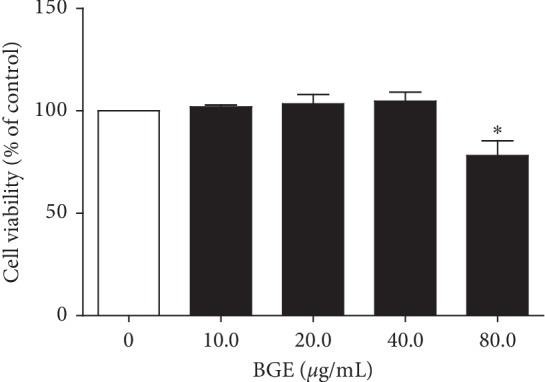
RAW264.7 macrophages were treated with increasing doses of BGE (0, 10, 20, 40, and 80 *μ*g/mL) for 20 h, and cell viability was determined by CCK8 assay. Data represent the mean ± SD of three independent experiments. ^*∗*^*P* < 0.05, compared with the control group.

To test the toxicity of BGE, the CCK-8 assay was used to assess the survival of RAW264.7 cells. As shown in Figure 4, 80 *μ*g/mL BGE reduced the cell survival by 22%, whereas 10 *μ*g/mL, 20 *μ*g/mL, and 40 *μ*g/mL BGE had no significant effect on cell viability. Therefore, we carried out in vitro experiments using these concentrations.

### 3.5. Inhibitory Effects of BGE on NO Production and Expression of iNOS and COX-2 Protein in RAW264.7 Cells

**Figure 5:**
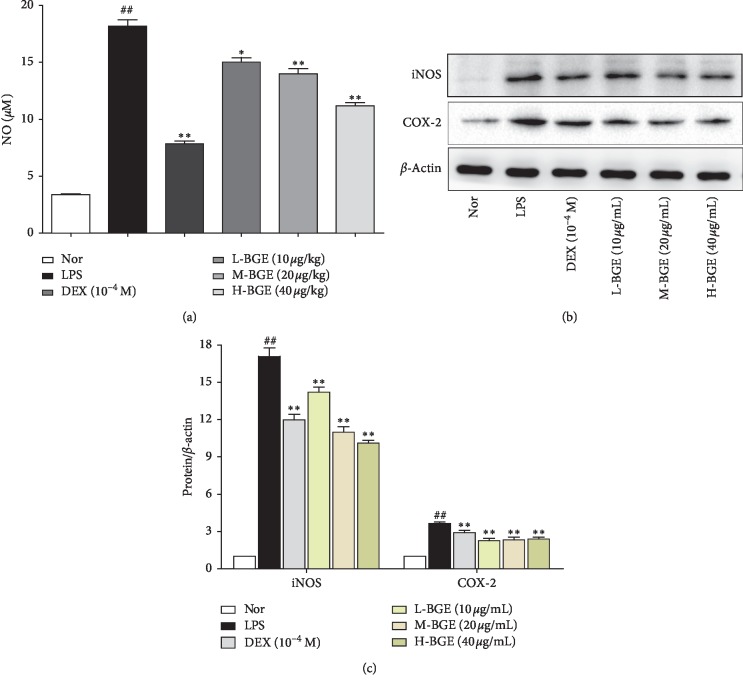
The effect of BGE on NO secretion and inflammatory protein expression. (a) RAW264.7 mouse macrophage was treated with BGE (10, 20, and 40 *μ*g/mL and LPS (1 *μ*g/mL)) for 24 h, and LPS-induced NO production levels were determined by the NO assay kit. Data represent the mean ± SD of three independent experiments. (b) Immunoblot analysis of expression of iNOS and COX-2 in RAW264.7 mouse macrophage treated with BGE (10, 20, and 40 *μ*g/mL)and LPS (1 *μ*g/mL) for 24 h. (c) Quantitative analysis of gray value of iNOS and COX-2 was performed in several groups with *β*-actin as loading control. Data represent the mean ± SD of three independent experiments. ^##^*P* < 0.01, compared with the normal group. ^*∗∗*^*P* < 0.01, ^*∗*^*P* < 0.05, compared with the LPS group.

As shown in Figure 5(a), BGE inhibited the increase in NO secretion induced by LPS. To explore the molecular mechanism by which BGE inhibits NO production, we further examined the effect of BGE on the expression of iNOS and COX-2. As shown in Figures 5(b) and 5(c), LPS stimulation upregulated iNOS and COX-2 protein expression in RAW264.7 cells (*P* < 0.01). However, BGE significantly inhibited the expression of iNOS and COX-2 proteins induced by LPS (*P* < 0.01).

### 3.6. Effect of BGE on Phosphorylation of Mitogen-Activated Protein Kinase in CFA Rats and in LPS-Stimulated RAW264.7 Cells

**Figure 6:**
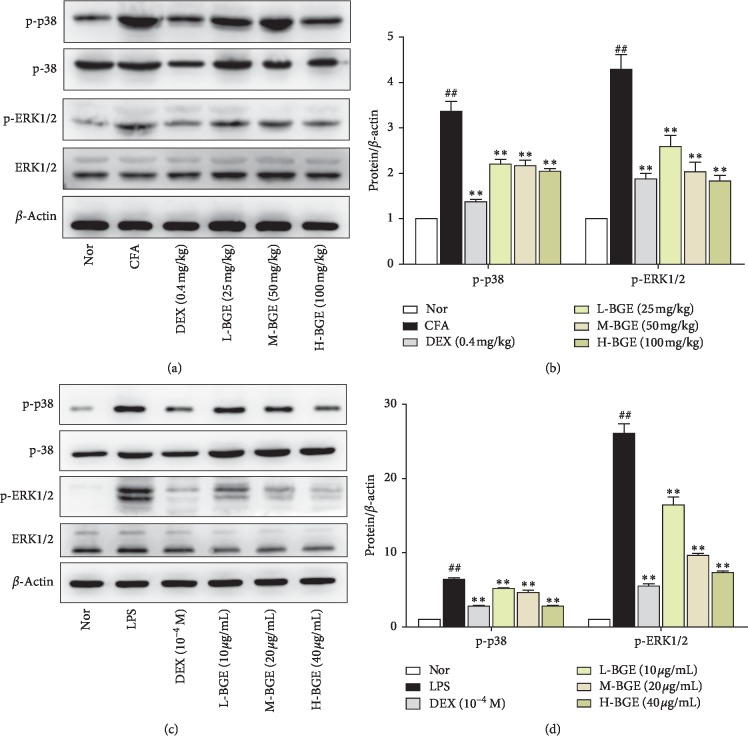
Effects of different doses of BGE on the MAPK signaling pathway. (a) Representative expression of p-38, p-p38, ERK, and p-ERK obtained from ankle joints of several groups was detected by western blot analysis. (b) Quantitative analysis of gray value of p-p38 and p-ERK was performed in several groups with *β*-actin as loading control. Data represent the mean ± SD of three independent experiments. (c) Immunoblot analysis of expression of p-38, p-p38, ERK and p-ERK in RAW264.7 mouse macrophage treated with BGE (10, 20, and 40 *μ*g/mL)and LPS (1 *μ*g/mL) for 24 h. (d) Quantitative analysis of gray value of p-p38 and p-ERK was performed in several groups with *β*-actin as loading control. Data represent the mean ± SD of three independent experiments. ^##^*P* < 0.01, compared with the normal group. ^*∗∗*^*P* < 0.01, ^*∗*^*P* < 0.05, compared with the CFA/LPS group.

As shown in Figures 6(a) and 6(b), after complete Freund's adjuvant injection, phosphorylated p38 and ERK of joint tissues in CFA group were significantly higher than those in the normal group (*P* < 0.01). BGE significantly inhibited the phosphorylation of p38 and ERK in the joint tissues of CFA rats (*P* < 0.01). Meanwhile, in vitro data confirmed that BGE can reverse the enhanced p38 and ERK phosphorylation induced by LPS in RAW264.7 cells (*P* < 0.01) (Figures 6(c) and 6(d)). Therefore, both animal and in vitro data indicate that BGE can inhibit phosphorylation of the MAPK signaling pathway.

## 4. Discussion

RA is a chronic systemic autoimmune inflammatory disease characterized by extensive connective tissue inflammation of the joint synovium, heart, lung, skin, and blood vessels [31]. The main clinical symptoms of RA are chronic inflammatory lesions of symmetrical multiple joints, which are characterized by pain, swelling, and decreased function of the joints. As the disease progresses, the synovial membrane, cartilage, and bone are gradually destroyed, eventually leading to joint deformity [32]. Since the etiology and pathogenesis of RA remain unclear, there is no effective treatment in clinic.

CFA-induced arthritis rats are widely used to screen for the treatment of rheumatoid arthritis drugs. CFA rats are closest to human rheumatoid arthritis in terms of pathogenesis, immunity, and genetic characteristics [33]. After rat modeling, the soft tissue swelling and the narrowing of the joint space were observed by X-ray, accompanied by an increase in the level of cytokines in the serum. Histological observation showed articular vasospasm formation, synovial hyperplasia, and inflammatory cell exudation. These results indicate that the rat model was successful. Compared with the model group, BGE can significantly alleviate the severity of RA, evidenced by inhibition of paw swelling, inflammatory cell exudation, and synovial cell proliferation. In addition, BGE can reduce the level of serum proinflammatory cytokines, which proves that BGE can effectively alleviate the progression of arthritis in vivo. In terms of drug safety, BGE treatment by intragastric administration did not cause death in rats, and the body weight of the rats were higher than those of the model group, which indicate that BGE has high safety in application. However, rats treated with dexamethasone showed a significant decrease in body weight, which was associated with increased catabolism in the body (Figure 1(b)).

As a central cell that initiates inflammatory mediators in vivo, macrophages play an important role in the pathogenesis of RA [34]. Many activated macrophages accumulate on the synovial and cartilage surfaces. Activated macrophages produce a number of proinflammatory mediators, including the secretion of TNF-*α* and IL-6, which cause fibroblast-like synoviocytes (FLS) to get activated and proliferated. At the same time, macrophages release tissue degrading enzymes, which cause the destruction of cartilage and bone. Clinical studies have shown that the number of macrophages in the inflammatory synovium is positively correlated with the severity of RA [35]. Therefore, we performed an in vitro anti-inflammatory assay in RAW264.7 mouse macrophages.

NO is a highly reactive oxidative-free radical in the body that is produced by endogenous L-arginine catalyzed by iNOS [36]. During the pathogenesis of RA, NO is directly involved in the inflammatory response and joint damage process. Abnormally elevated NO can lead to excessive blood vessel expansion and increased vascular exudation while also promoting the release of proinflammatory cytokines (TNF-*α*, IL-6, and IL-1*β*). However, cytokines (TNF-*α* and IL-1*β*) can also induce the synthesis of iNOS to increase the level of NO, which promotes each other and forms a vicious circle [37]. Excessive NO can react with free radicals to form ONOO-, which can cause tissue damage and joint lesions [38]. Experimental data indicate that BGE can significantly inhibit NO secretion in LPS-induced RAW264.7 macrophages. Moreover, we confirmed that BGE can downregulate the expression of iNOS and COX-2 from the level of protein expression, which is consistent with the reduction of NO synthesis (Figures 5(b) and 5(c)). COX-2 is an inducible oxidase and an important key enzyme for the synthesis of prostaglandins. It is usually expressed together with iNOS in inflammatory cells [39]. In addition, ELISA assay confirmed that BGE reduces the level of LPS-induced proinflammatory cytokines, thereby admitting its potential to regulate the inflammatory and oxidation status of macrophages.

Many proinflammatory cytokines can participate in the regulation of immune cell differentiation, mediate the body's immune response, and play an important role in the pathogenesis of the disease [30]. Among these proinflammatory factors, TNF-*α*, IL-1*β*, and IL-6 are most involved in the development of RA. Studies have confirmed that TNF-*α* is likely to be an upstream promoter in the process of rheumatoid arthritis inflammation. On the one hand, the expression levels of IL-1*β*, IL-6, IL-8, and granulocyte-macrocellular colony-stimulating factor in serum were downregulated by the TNF-*α* antibody [40]. On the other hand, overexpression of TNF-*α* leads to degradation of articular cartilage by promoting the expression of metalloproteinases, proteoglycans, and type II collagen [41]. IL-1*β* can downregulate the expression level of tissue inhibitor of metalloproteinase-3 and upregulate the expression levels of interstitial metalloproteinase-1, interstitial metalloproteinase-3, and IL-6. The overall effect of IL-1*β* is to promote the degradation of extracellular matrix and the destruction of joint cartilage [42]. Both IL-1*β* and TNF-*α* can induce IL-6 production by fibroblasts, chondrocytes, and synovial macrophages. IL-6 can promote the formation of various autoantibodies in plasma cells, causing humoral immune regulation disorders [43]. Our results demonstrate that BGE can inhibit the secretion of TNF-*α*, IL-6 in LPS-induced RAW264.7 macrophages (Figure 3(a)), and serum levels of TNF-*α*, IL-1*β*, and IL-6 in CFA rats (Figure 3(b)). Therefore, the BGE can exert an anti-arthritic effect by inhibiting the production of cytokines.

The main function of MAPK is to transmit the inflammatory stimuli to the nucleus, which mediates chronic inflammation and joint damage in RA by activating downstream inflammatory factors. ERK and p38 are two major members of the MAPK family that are closely related to the maintenance of pathological state of RA and progression of the disease [8]. Studies have confirmed that the p38MAPK pathway is strongly associated with inflammatory response. The p38MAPK inhibitor (SB 203580) not only inhibits the secretion of IL-1 and TNF-*α* but also reduces the levels of NO, COX-2, metalloproteinase-1, and metalloproteinase-3 induced by IL-1 [9]. The ERK pathway mediates the transmission of mitotic signals to the nucleus, thereby regulating the cell proliferation. It can also promote the secretion of various cytokines and the formation of pannus [10]. In this experiment, administration of BGE significantly inhibited phosphorylation of ERK and p38 in LPS-induced RAW264.7 cells (Figures 6(a) and 6(b)) and CFA rat joints (Figures 6(c) and 6(d)). Therefore, this result suggests that the antiarthritis underlying mechanism of BGE may be via inhibiting the expression of the MAPK pathway.

In vitro experimental results indicate that BGE reduces cytokine secretion levels by downregulating p38 and ERK phosphorylation in LPS-treated RAW264.7 macrophages. In addition, BGE can inhibit the synthesis of NO by downregulating the expression levels of iNOS and COX-2. In vivo, BGE can reduce the swelling of the paw and the secretion of serum cytokines. Through pathological evaluation, BGE effectively reduces the exudation of inflammatory cells in the joints, the proliferation of synovium, and the formation of pannus. In summary, BGE can be used as an alternative treatment for RA and its mechanism may be that BGE reduces cytokine exudation by inhibiting phosphorylation of p38 and ERK in the MAPK inflammatory pathway. At present, eight kinds of phenolic compounds have been separated from the ethanol extract of *Gastrodia elata* by column chromatography. According to the previous studies, it is speculated that the anti-inflammatory effect may be a phenolic compound containing a C-4 hydroxyl group and a C-3 methoxy group [44]. However, our further experiments are required to screen out which molecular compounds in the *n*-butanol extract exert an anti-arthritic effect.

## Data Availability

The data used to support the findings of this study are available from the corresponding author upon request.
